# *Plantago asiatica* L. Ameliorates Puromycin Aminonucleoside-Induced Nephrotic Syndrome by Suppressing Inflammation and Apoptosis

**DOI:** 10.3390/nu9040386

**Published:** 2017-04-14

**Authors:** Min Chul Kho, Ji Hun Park, Byung Hyuk Han, Rui Tan, Jung Joo Yoon, Hye Yoom Kim, You Mee Ahn, Yun Jung Lee, Dae Gill Kang, Ho Sub Lee

**Affiliations:** 1 Hanbang Body-fluid Research Center, Wonkwang University, 460 Iksandae-ro, Iksan, Jeonbuk 54538, Korea; shadowzetx@hanmail.net (M.C.K.); jihuncjstk@naver.com (J.H.P.); arum0924@nate.com (B.H.H.); tanrui@hanmail.net (R.T.); morality16@hanmail.net (J.J.Y.); hyeyoomc@naver.com (H.Y.K.); aum2668@naver.com (Y.M.A.); shrons@wku.ac.kr (Y.J.L.); 2 College of Oriental Medicine and Professional Graduate School of Oriental Medicine, Wonkwang University, 460 Iksandae-ro, Iksan, Jeonbuk 54538, Korea

**Keywords:** nephrotic syndrome, plantago asiatica, inflammation, apoptosis, ascites

## Abstract

Objective: Nephrotic syndrome, a kidney disease with a variety of causes, is mainly characterized by heavy proteinuria, hypoproteinemia, and ascites. This study was designed to evaluate the underlying mechanism of action of *Plantago asiatica* L. (PAL) in treating nephrotic syndrome induced by puromycin aminonucleoside. Methods: PAL has been used in Asia as a traditional medicine and dietary health supplement. Sprague-Dawley (SD) rats were intravenously injected with puromycin aminonucleoside (75 mg/kg/day), then treated with either Losartan (30 mg/kg/day) or PAL (200 mg/kg/day) by oral gavage for seven days. Results: PAL significantly decreased ascites, proteinuria level, and plasma lipid parameters. In addition, treatment with PAL attenuated histological damage and hypoalbuminemia. Treatment with PAL also restored podocin expression and reduced inflammation markers such as intracellular adhesion molecules (ICAM-1), monocyte chemotactic protein-1 (MCP-1), tumor necrosis factor alpha (TNF-α) and high-mobility group box-1 (HMGB1). Lower expression levels of the apoptosis markers Bax, caspase-3 and capase-9 were documented in SD rats receiving PAL. PAL also significantly decreased the phosphorylation levels of MAPKs such as ERK, JNK and p38. Conclusion: As a multifunctional agent, PAL has a renoprotective effect in nephrotic syndrome rat models. The anti-inflammatory and anti-apoptotic properties, along with reductions in hyperlipidemia and ascites, represent important therapeutic effects. These results indicate that *Plantago asiatica* is likely to be a promising agent in the treatment of nephrotic syndrome.

## 1. Introduction

Nephrotic syndrome (NS), a kidney disease with a variety of causes, is mainly characterized by heavy proteinuria, hypoproteinemia and ascites [1]. NS patients suffer from severe complications such as hypercoagulation, thrombotic symptoms, and disorders of metabolism [2].

The pathogenesis of NS is complex and not entirely clear, involving the circulatory and metabolic systems among others. Many studies suggest that renal inflammation, oxidative stress and apoptosis are typically associated with the development and progression of NS pathological features [3,4,5]. Various factors associated with the progression of glomerulonephritis, nephrotoxicity, and other renal disorders, such as chemokines and adhesion molecules, are secreted by resident renal cells. These factors attract and induce infiltration by circulating inflammatory cells [6]. In addition, pro-inflammatory cytokines increase synthesis of other inflammatory molecules and trigger cell apoptosis [7]. Apoptosis caused by environmental and intrinsic stimuli results in a loss of resident kidney cells, and subsequent development of kidney injury [8]. Therefore, in proteinuric renal disorders, such as nephrotic syndrome and chronic renal disease, inflammation and apoptosis are closely linked to, and implicated in, renal injury [9].

Traditional herbal medicines have been used, with apparent safety and efficacy, to treat and alleviate various complex refractory diseases, such as cancer and nephrotic syndrome [10,11,12,13]. *Plantago asiatica* L. (PAL) has been used for more than 2000 years in Asia as a traditional medicine and dietary health supplement [14]. In addition, analysis certification of PAL was approved by permission of the Korea food and drug administration (KFDA, PLAS2014). According to this certificate of analysis, Geniposidic acid and acteoside are main ingredients of PAL. It has previously been reported that extract of PAL was found to have anti-depressant, hypoglycemic, anti-oxidant, and lipid metabolism regulating effects [15,16,17]. However, renoprotective effects of PAL have not been reported thus far. Thus, the aim of the present study was to investigate the renoprotective effects of PAL in nephrotic syndrome in terms of renal function, inflammation, and apoptosis.

## 2. Materials and Methods

### 2.1. Preparation of Seed of Plantago asiatica L.

Dried raw seed of *Plantago asiatica* L. (PAL) herb was purchased from the Herbal Medicine Co-operative Association, Iksan, Jeonbuk Province, Korea. PAL seed (200 g) was soaked in double-distilled water, then boiled with 1.5 L of distilled water at 100 °C for 2 h. The extract was filtered through Whatman No. 3 filter paper (Whatman International Ltd., Maidstone, UK) and centrifuged at 990× *g* for 20 min at 4 °C. Supernatant was concentrated using a rotary evaporator and the resulting extract (13.12 g) was lyophilized using a freeze-drier. The extract voucher specimen (No. HBJ101) was kept at 4 °C and deposited in the herbarium of the Professional Graduate School of Oriental Medicine, Wonkwang University (Iksan, Jeonbuk, Korea). The extract was dissolved in distilled water before use.

### 2.2. Drugs and Chemicals

The puromycin aminonucleoside for injection was purchased from Enzo Life Sciences, Inc. (Farmingdale, NY, USA). Losartan, used as the positive control drug, was purchased from Santa Cruz Biotechnology (Santa Cruz, CA, USA).

### 2.3. Animal Experiments and Treatment Protocol

All experimental protocols involving the use of animals were conducted in accordance with National Institutes of Health (NIH) guidelines and approved by the Institutional Animal Care and Utilization Committee for Medical Science of Wonkwang University (WKU12-12). Eight-week-old male Sprague-Dawley (SD) rats (weighing 170–200 g) were obtained from Samtako (Osan, Korea). Rats were kept in a room with automatic maintenance of temperature (23 ± 2 °C), humidity (~50%–60%), and 12-h light/dark cycle throughout the experiments. During the experiments, all animals were given standard rat chow diet *ad libitum*. Rats were randomly divided into four groups: Control group; a puromycin aminonucleoside-treated NS group; a NS plus Losartan (30 mg/kg/day) treated group; and a NS plus PAL (200 mg/kg/day) treated group. Rats were intravenously injected with puromycin aminonucleoside (75 mg/kg/day, dissolved in saline). The control group was injected with saline only. Rats were administered either distilled water (Control and NS groups), Losartan or PAL by an oral gavage method once daily for 7 days. During the experiments, all animals were housed separately in a metabolic cage and 24-h urine samples were collected before sacrifice. On day 7, the end of the study period, all rats were sacrificed for blood and renal tissues. Blood samples were collected and centrifuged to separate plasma at 600× *g* for 15 min at 4 °C. The weight of the ascitic fluid was measured by inserting a weighed tissue paper into the abdomen to absorb the fluid before reweighing it.

### 2.4. Measurement of Urinary Protein Excretion

For urinary protein analysis, each animal was housed separately in a metabolic cage and 24-h urine samples were collected after puromycin aminonucleoside injection. Urinary protein concentrations were determined using Bradford Dye Reagent (Bio-Rad, München, Germany).

### 2.5. Measurement of Plasma Biochemical Parameters

Plasma total cholesterol (T-Cho), triglyceride (TG), low-density lipoprotein cholesterol (LDL-c), blood urea nitrogen (BUN), total protein (T-pro) and albumin (Alb) levels were measured using commercially available kits (ARKRAY, Inc., Minami-ku, Kyoto, Japan). Plasma creatinine was measured by a colorimetric method using a spectrophotometer [18] (Miloton Roy, Rochester, NY, USA). Pre-chilled EDTA-coated tube was used as an anticoagulant (BD Vacutainer, BD Franklin Lakes, NJ, USA).

### 2.6. Histopathological Staining of Renal Tissues

For morphological staining, isolated kidney tissues were fixed with 10% formalin, dehydrated in graded alcohol, and embedded in paraffin. Sections measuring 5 μm were cut and stained with periodic acid-Schiff (PAS) reagent. All slides were evaluated by the same pathologist, who was unaware of the nature of the experimental groups. Images of kidney sections were taken with Axiovision 4 Imaging/Archiving software. For quantitative analysis, the average score of 10–20 randomly selected areas was calculated using image analysis software. Images were analyzed for protein cast formation using the Image J (NIH, Bethesda, MD, USA) program to select and quantify PAS-stained areas as a percentage of the total area of each image.

### 2.7. Immunohistochemical Staining of Renal Tissues

Kidney samples were fixed with 10% formalin and embedded in paraffin. After antigen retrieval to suppress endogenous peroxidase activity, slides were immersed in 3% hydrogen peroxide for 10 min. Sections were incubated with the appropriate primary in humidified chambers overnight at 4 °C. Viewing was performed using the 3,3′-diaminobenzidine (DAB; Novex^®^, San Diego, CA, USA) substrate-chromogen system, with hematoxylin (Zymed, San Francisco, CA, USA) counterstaining. Image J software was used for quantitative analysis.

### 2.8. Western Blot Analysis

Renal tissue homogenates (40 μg of protein) were separated using 10% SDS-polyacrylamide gel electrophoresis and transferred to nitrocellulose paper membranes. Blots were then blocked by 5% BSA powder in Tris-buffered saline (TBS) for 1 h, and incubated with appropriate primary antibodies to Podocin, ICAM-1, MCP-1, HMGB-1, TNF-α, Bcl-2, Bax, Caspase-3, Caspase-9, phospho-p38, p38, phospho-ERK1/2, ERK1/2, phosphor-JNK, JNK or β-Actin (Santa Cruz Biotechnology, Santa Cruz, CA, USA). Subsequently, the membrane was incubated with a secondary goat anti-rabbit IgG or goat anti-mouse IgG antibody conjugated to horseradish peroxidase (Enzo Life Sciences, Farmingdale, NY, USA). The bands were visualized with enhanced chemiluminescence (Amersham, Buckinghamshire, UK). Protein expression levels were determined by analyzing signals captured on the nitrocellulose membranes using the ChemiDoc image analyzer (Bio-Rad, Hercules, CA, USA).

### 2.9. Statistical Analysis

Results were expressed as mean ± SE, and the data were analyzed using one-way ANOVA followed by student’s *t*-test using SIGMAPLOT (Sigma plot ver. 10.0) to determine any significant differences. Significance was set at *p* < 0.05.

## 3. Results

### 3.1. Effects of PAL on Proteinuria and Ascites

**Figure 1:**
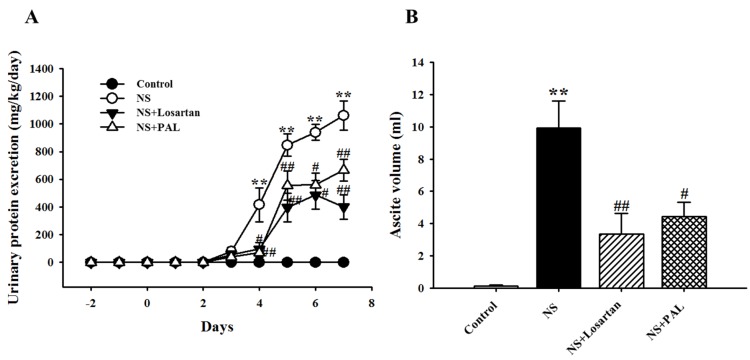
Effects of *Plantago asiatica* L. (PAL) on urinary protein excretion at various time points (**A**) and ascites (**B**). Values were expressed as mean ± SE (*n* = 7). ** *p* < 0.01 versus Control; ^#^ *p* < 0.05, ^##^ *p* < 0.01 versus NS.

Proteinuria is an important characteristic of puromycin aminonucleoside-induced NS in rats. As shown in Figure 1A, 24-h urinary protein excretion progressively increased following puromycin aminonucleoside injection. Proteinuria levels in the NS group peaked on day 7 compared to the Control group. However, the PAL-treated group exhibited significantly decreased proteinuria levels on day 7 (*p* < 0.01). Similarly, treatment with losartan produced similar results to those seen in the PAL group. Ascites is another important characteristic of puromycin aminonucleoside-induced rat NS. As shown in Figure 1B, the NS group had significantly increased ascites volume compared with the Control group. Both the PAL- and Losartan-treated groups had significantly decreased ascites volume.

### 3.2. Effect of PAL on Plasma Parameters and Renal Function

The NS group exhibited increases in T-Cho, TG and LDL-c levels, which are clinical data of nephrotic syndrome. However, the PAL-treated group exhibited significantly lower levels of these markers (Table 1, *p* < 0.05). Treatment with losartan yielded similar results to those of the PAL-treated group. As indicated in Table 2, compared with the Control group, the NS group exhibited increased plasma BUN and creatinine levels. However, results from the PAL-treated group demonstrated attenuation of these increased levels (*p* < 0.01). In addition, intravenous puromycin aminonucleoside decreased T-pro and Alb levels. However, the PAL-treated group had significantly increased T-pro and Alb levels (*p* < 0.05). Again, treatment with losartan yielded similar results. These results indicated that PAL could improve renal function and reduce hyperlipidemia in puromycin aminonucleoside-induced NS rats.

### 3.3. Effect of PAL on Renal Morphology

**Figure 2:**
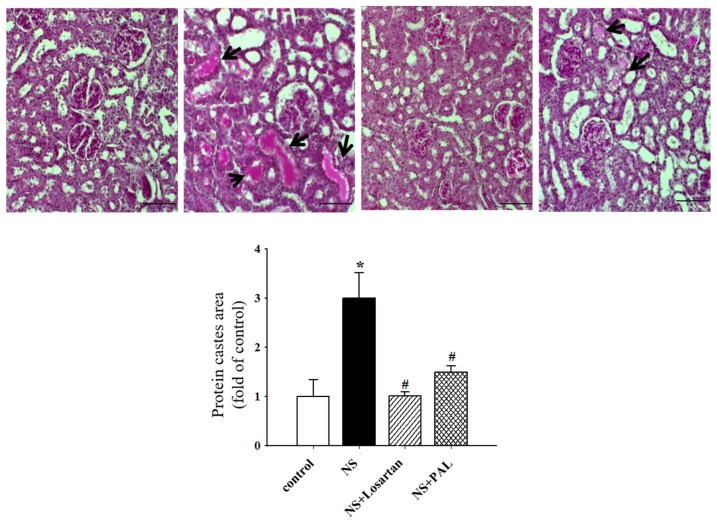
Effects of treatment of PAL on renal morphology. Representative photomicrographs of PAS (Periodic acid-chiff)-stained tissues (magnification ×200). The bottom panels represent quantitative assessments of protein cast area. Protein casts in the distal tubules are indicated by black arrows in the pictures. Values were expressed as mean ± SE (*n* = 7). * *p* < 0.05 versus Control.; ^#^ *p* < 0.05 versus NS.

There were no histopathological changes in the glomerular structures in any of the groups (Figure 2). In parallel with the proteinuria, the puromycin aminonucleoside-treated rats exhibited marked pathological lesions characterized by abundant protein exudation in renal tubular lumens and marked interstitial inflammatory cell infiltration. However, treatment with PAL attenuated the pathological damage to some degree, by reducing inflammatory cell infiltration and protein cast formation. In addition, pathological scores were significantly decreased. Treatment with losartan yielded similar results to PAL. These results implied that PAL could alleviate pathological damage to the kidney in puromycin aminonucleoside-induced NS rats.

### 3.4. Effect of PAL on Podocyte Injury

**Figure 3:**
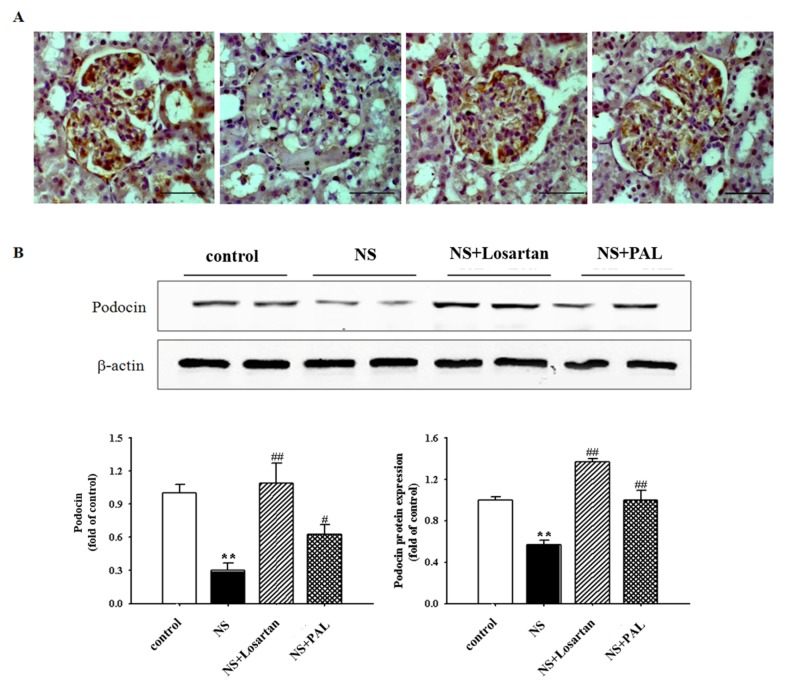
Effects of treatment of PAL on renal podocin expression. The top panels show immunohistochemistry staining (magnification ×400) (**A**) and western blot (**B**) of renal cortical tissue. The bottom panels represent quantitative assessments of podocin expression. Values were expressed as mean ± SE (*n* = 7). ** *p* < 0.01 versus Control.; ^#^ *p* < 0.05, ^##^ *p* < 0.01 versus NS.

Podocin is specifically localized to the podocyte foot process, which functions to maintain glomerular filtration permeability. The NS group exhibited significantly decreased expression of podocin in western blot and immunohistochemistry assays compared with the Control group (Figure 3). However, treatment with PAL significantly recovered the expression of podocin levels as measured by both techniques (*p* < 0.01). Treatment with losartan yielded similar results. These results suggest that PAL could enhance podocyte foot processes by recovering the expression of podocin in puromycin aminonucleoside-induced NS rats.

### 3.5. Effect of PAL on Inflammatory Markers in the Kidney

**Figure 4:**
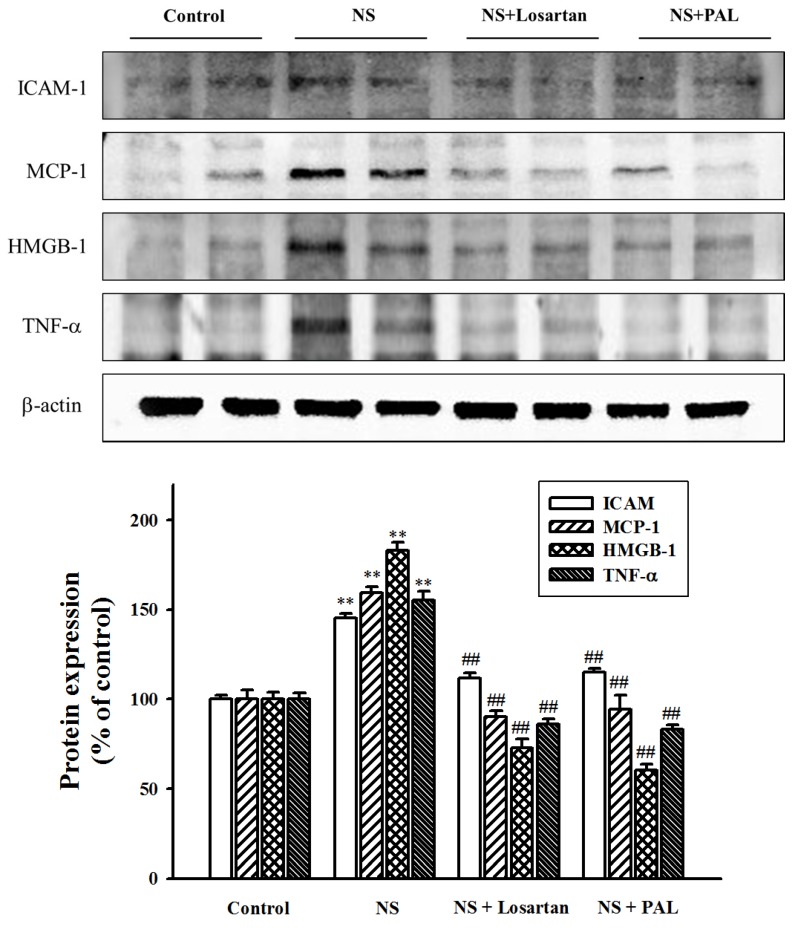
Effect of PAL on the expression of inflammation markers in renal tissues. The whole kidney extracts were prepared, and ICAM-1, MCP-1, HMGB-1 and TNF-α were analyzed by western blot analysis. Each electrophoretogram represents the results from three individual experiments. ** *p* < 0.01 versus Control.; ^##^ *p* < 0.01 versus NS.

Proteinuria induces activation of inflammatory markers in proximal tubular cells [19,20]. In this present study, we investigated inflammatory markers such as ICAM-1, HMGB1, MCP-1 and TNF-α. The NS group demonstrated progressively increasing expression of these markers compared with the Control group. However, treatment with PAL significantly decreased the expression of these markers (Figure 4). Similarly, treatment with losartan resulted in decreased inflammatory markers. These results suggest that puromycin aminonucleoside-induced renal inflammation was mediated by increases in ICAM-1, HMGB1, MCP-1 and TNF-α. Urine protein levels, therefore, correlate with inflammatory marker levels. PAL may ameliorate renal inflammation directly by reducing proteinuria.

### 3.6. Effect of PAL on Apoptosis-Related Markers in Renal Tissues

**Figure 5:**
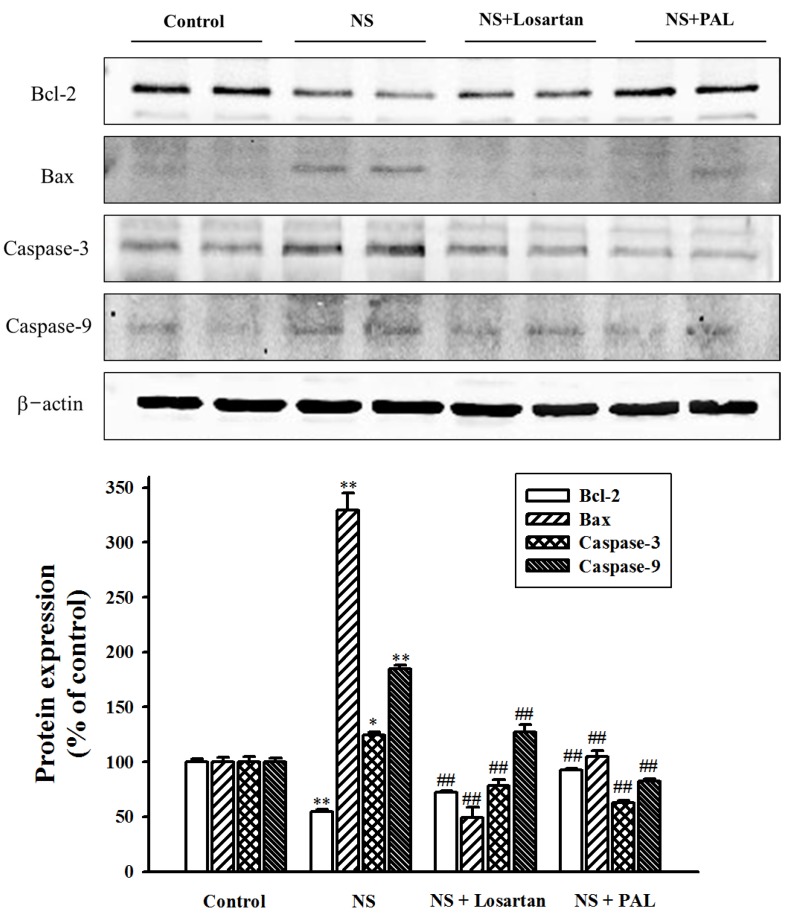
Effect of PAL on the expression of apoptosis-related markers in renal tissues. Whole-kidney extracts were assayed for Bcl-2, Bax, Caspase-2 and Caspase-9 by western blot analysis. Each electrophoretogram is representative of the results from three individual experiments. * *p* < 0.05, ** *p* < 0.01 versus Control.; ^##^ *p* < 0.01 versus NS.

Caspase-3, caspase-9, Bcl-2 and bax proteins direct cell apoptosis by way of intracellular and extracellular death signals. To facilitate investigation of the mode of action of PAL, concentrations of these apoptosis-related markers in the renal tissue were determined. The results showed that expression of caspase-3, caspase-9 and bax protein levels were significantly increased, whereas expression of Bcl-2 was significantly decreased in the NS group compared with the Control group. However, treatment with PAL down-regulated caspase-3, caspase-9 and bax levels, while recovering expression of Bcl-2 protein (Figure 5). Similar results were seen in the losartan-treated. These results suggest that puromycin aminonucleoside-induced renal apoptosis is due to both increases in caspase-3, caspase-9 and bax levels, as well as reduced bcl-2 protein levels. These are direct targets for PAL in mediating the reduction of apoptosis.

### 3.7. Effect of PAL on MAPK Signal Pathway in Renal Tissues

**Figure 6:**
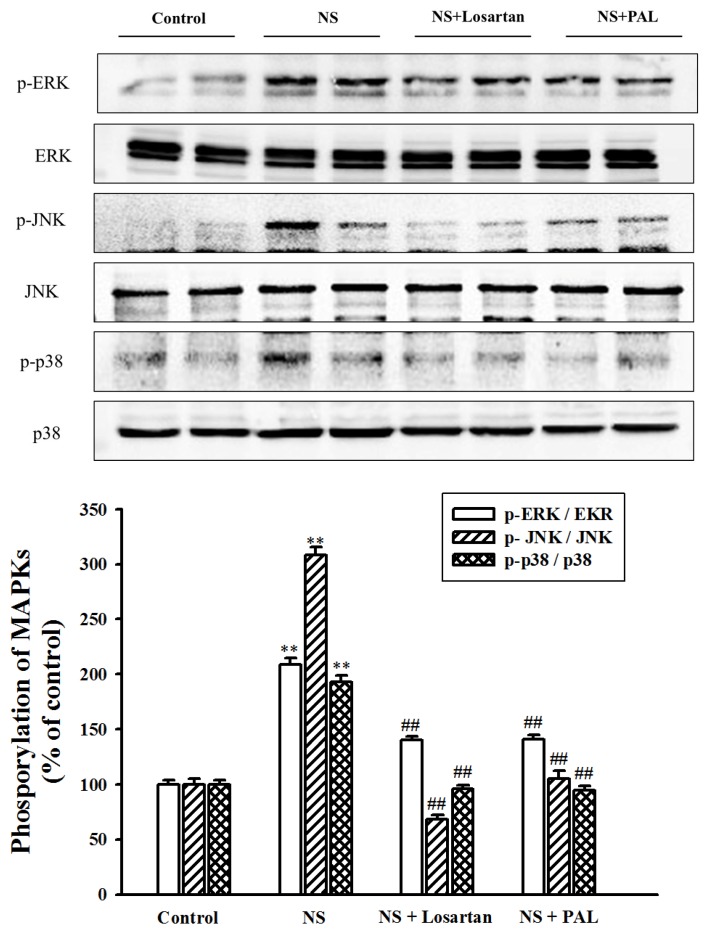
Effect of PAL on the expression of MAPK in renal tissues. MAPKs were detected by specific antibodies and compared to the corresponding signals from phosphorylated MAPKs. Whole-kidney extracts were assayed for phosphorylated MAPKs western blot analysis. Each electrophoretogram is representative of the results from three individual experiments. ** *p* < 0.01 versus Control.; ^##^ *p* < 0.01 versus NS.

Activation of MAP kinases, such as ERK, JNK and p38, is associated with renal injury involving cellular changes such as cell proliferation, inflammation, and apoptosis in renal cells [21]. To determine whether MAP kinases are involved in the progression of puromycin aminonucleoside-induced nephrotic syndrome, we examined the phosphorylated form of each MAP kinase. Our results showed that the puromycin aminonucleoside-injected group exhibited increased levels of the phosphorylated forms of ERK, JNK, and p38. However, treatment with PAL suppressed these increases (Figure 6). Treatment with losartan yielded similar results. In addition, the results demonstrated no increase in total ERK, JNK and p38 levels in any group.

## 4. Discussion

Nephrotic syndrome (NS) is one of the most common glomerular diseases, which include proteinuria, hypoalbuminemia, hyperlipidemia and edema [3]. Despite the advanced technology and drugs that have been or are currently being developed to treatment and treat nephrotic syndrome, existing pharmaceutical treatments are not sufficient to treat NS patients from developing renal diseases [22]. Therefore, it is necessary to seek out other effective drugs to target and treat NS.

The present study used a single puromycin aminonucleoside injection method to induce a model NS, the features of which are similar to those of the clinical syndrome. This model has been widely used in previous studies [22,23,24]. Typically, this model results in histological abnormalities of glomerular epithelial and endothelial cells. Many previous studies have demonstrated that proteinuria is accompanied by kidney lesions. Increased inflammation and apoptosis may be the underlying mechanism of these changes [7,8,9].

Maintenance of glomerular filtration by podocytes depends on podocin, nephrin and CD2-associated proteins, which are known to be involved in maintaining the structural integrity of the slit diaphragms [25]. Thus, podocyte apoptosis is an important factor in puromycin aminonucleoside-induced nephrosis and the progression of focal segmental glomerulosclerosis [26]. Results of the present study, showed that treatment with PAL significantly decreased 24-h proteinuria and edema in puromycin aminonucleoside-induced nephrosis. In addition, the increased podocin expression, higher plasma levels of albumin and protein and decrease in protein cast formation in our morphological study were correlated with the inhibition of proteinuria. These results provide evidence that PAL may represent a novel therapy for NS.

As inflammation and apoptosis promote progression of renal injuries in a puromycin aminonucleoside-induced NS model, we hypothesize that the renoprotective function of PAL may be partly due to its anti-inflammatory and anti-apoptosis effects. Intracellular adhesion molecules (ICAM-1), monocyte chemotactic protein-1 (MCP-1), tumor necrosis factor alpha (TNF-α) and high-mobility group box-1 (HMGB1) are pro-inflammatory cytokines, chemokines and/or proteins. They are strongly associated with the activation and progression of renal injury and inflammation, as well as triggering cell death in various kidney cells, including tubular epithelial cells, podocytes, mesangial cells and endothelial cells [27]. Several studies have reported that increases in these factors are associated with increased proteinuria and leakage of albumin and proteins in patients with renal diseases [19,20]. The present study demonstrated that PAL ameliorated increases in pro-inflammatory cytokines, chemokines and proteins. This suggests that PAL may inhibit puromycin aminonucleoside-induced nephrosis.

Hyperlipidemia is also one of the main features of puromycin aminonucleoside-induced NS in rats [22,23,24]. Several reports have suggested that apoptosis of the tubular epithelium is probably attributable to the impact of proteinuria and lipid toxicity. In addition, albumin bound to fatty acids in urine is reabsorbed by proximal tubular epithelial cells and causes apoptosis and renal damage [28,29]. The present study showed that treatment with PAL significantly decreased plasma levels of T-Cho, TG, and LDL-c. Therefore, our study deduced that the reduction of apoptosis by treatment with PAL may be partially due to its down-regulation and moderation of lipid levels, thus decreasing urinary protein levels.

To clarify the anti-apoptotic mechanism of PAL, our study investigated the influence of PAL on the expression of major apoptosis regulating factors such as caspase-3, caspase-9, Bcl-2 and bax. Caspases and bax are major apoptosis effectors, being considered the protein triggers for cell death. Proteins of the Bcl family, such as Bcl-2, play a pivotal anti-apoptotic role, maintaining cellular homeostasis through suppression of the endoplasmic reticulum. In addition, they have the ability to inhibit pro-apoptotic mechanisms and promote cell survival [30,31]. The present study showed that PAL could down-regulate apoptotic factors such as caspase-3, caspase-9 and bax, as well as up-regulating anti-apoptotic factors such as Bcl-2. In this way, PAL may act as an effective anti-apoptotic agent.

Renal function is closely correlated with urinary protein excretion level and is a direct reflection of progression of renal disease. The concentrations of plasma BUN and creatinine, which are important indices reflecting renal function, depend on the glomerular filtration rate (GFR). Renal dysfunction reduces their filtration, and therefore levels rise [32]. In the present study, our results showed that levels of plasma creatinine and blood urea nitrogen were increased and levels of plasma albumin were decreased by injection of puromycin aminonucleoside, indicating that renal filtration function was impaired by puromycin aminonucleoside. However, treatment with PAL counteracted these changes, indicating that PAL could enhance renal filtration in puromycin aminonucleoside-induced NS rats.

The mitogen-activated protein kinases (MAPKs) react to extracellular stimuli (mitogens) and control various cellular activities including gene expression, mitosis, differentiation, cell survival and apoptosis [33] MAP kinases regulate important cellular functions through the activation of specific signal transduction pathways from the cell surface to the nucleus [34]. It has been reported that these kinases are activated by numerous factors implicated in the pathogenesis of various renal injuries, including cytokines and cellular stress [35]. In addition, MAPKs also control cellular responses to cytokines and stress, both of which are related to inflammation and the immune system. In addition, they play a critical role in modulating the NF-κB pathway [36,37]. Recently, several studies have suggested that the MAP kinase family is activated in the kidney, contributing to progressive renal injury in, for example, ischemia-reperfusion, modeled diabetes and puromycin aminonucleoside-induced nephropathy [21,38]. In the present study, the results showed that PAL decreased the expression of MAP kinases such as ERK, JNK and p38. Therefore, our study demonstrated that the regulating of MAP kinase by treatment with PAL may inhibit signaling of inflammation and apoptosis in renal injury [39]. A major circulating factor in FSGS is soluble urokinase receptor suPAR, which has also a role as inflammatory mediator [40]. This molecule was not analyzed in our study but future investigations will address this.

There are some limitations from not providing the composition of PAL and comparing glomerular ultrastructure in this study. The next study will be focused on the validation of PAL by HPLC and glomerular function in NS animal models.

## 5. Conclusions

The present study demonstrates that treatment with *Plantago asiatica* L. effectively reduced proteinuria, edema, concentrations of plasma creatinine and blood urea nitrogen, treated glomerular morphological change, up-regulated the expression of podocin and inhibited activation of inflammatory and apoptotic factors. *Plantago asiatica* L. also decreased phosphorylation of MAPKs. These results suggest that *Plantago asiatica* L. could be a credible new curative therapeutic option to ameliorate renal dysfunction seen in the nephrotic syndrome.

## Figures and Tables

**Table 1:** Effect of treatment PAL on plasma lipids.

Groups	Control	NS	NS	
			Losartan	PAL
T-Cho (mg/dL)	63.4 ± 3.2	397.8 ± 2.3 **	258.2 ± 38.4 ^#^	301.8 ± 34.0 ^#^
TG (mg/dL)	97.6 ± 16.1	196.6 ± 3.4 **	352.3 ± 65.3 ^#^	355.2 ± 41.1 ^##^
LDL-c (mg/dL)	20.8 ± 3.6	171.2 ± 9.0 **	90.6 ± 20.9 ^##^	110.6 ± 22.0 ^#^

Values were expressed as mean ± SE (*n* = 7). ** *p* < 0.01 versus Control; ^#^ *p* < 0.05, ^##^ *p* < 0.01 versus NS. Abbreviations: T-Cho, total cholesterol; TG, triglyceride; LDL-c, low-density lipoprotein cholesterol.

**Table 2:** Effect of treatment PAL on renal functional parameters.

Groups	Control	NS	NS	
			Losartan	PAL
BUN (mg/dL)	13.7 ± 0.8	56.4 ± 5.5 **	33.9 ± 6.3 ^#^	30.8 ± 3.6 ^##^
T-pro (mg/dL)	5.6 ± 0.1	3.6 ± 0.1 **	4.8 ± 0.3 ^##^	4.2 ± 0.2 ^#^
Alb (mg/dL)	4.1 ± 0.1	1.1 ± 0.2 **	2.9 ± 0.4 ^##^	2.2 ± 0.3 ^##^
Plasma creatinine (mg/dL)	0.11 ± 0.01	0.25 ± 0.03 **	0.13 ± 0.02 ^##^	0.15 ± 0.01 ^##^

Values were expressed as mean ± SE (*n* = 7). ** *p* < 0.01 versus Cont.; ^#^ *p* < 0.05, ^##^ *p* < 0.01 versus NS. Abbreviations: BUN, blood urea nitrogen; T-pro, total protein; Alb, albumin.
